# Association of diabetic vascular complications with poor sleep complaints

**DOI:** 10.1186/s13098-016-0195-8

**Published:** 2016-12-08

**Authors:** Ling-Ling Meng, Ying Liu, Rui-Na Geng, Yun-Zhao Tang, Dai-Qing Li

**Affiliations:** 1 Key Laboratory of Hormones and Development (Ministry of Health), Tianjin Key Laboratory of Metabolic Diseases, Metabolic Diseases Hospital and Tianjin Institute of Endocrinology, Tianjin Medical University, Tongan Road 66, Heping District, Tianjin, 300070 China; 2 Third Division of Endocrinology Department, Hebei Cangzhou Central Hospital, Xinhua West Road 16, Yunhe District, Cangzhou, 061000 Hebei China

**Keywords:** Poor sleep complaints, Diabetic kidney disease (DKD), Neuropathy, Diabetic retinopathy (DR), Cardiovascular disease (CVD), Peripheral arterial disease (PAD)

## Abstract

**Background:**

Literatures reported that poor sleep complaints were associated with a great deal of health outcomes. However, there are few studies on the association of poor sleep complaints with diabetic vascular complications.

**Methods:**

Aiming on the association, a cross-sectional survey was conducted among 1220 diabetic patients in this study. Poor sleep complaints were composed of difficulty falling asleep, early final awakening, short sleep and long sleep. The diabetic vascular complications involved in the study were diagnosed according to the Standards of Medical Care in Diabetes (ADA 2016).

**Results:**

Our findings indicated that short sleep remained independently associated with diabetic kidney disease (DKD) (OR > 1, P < 0.05) after the adjustments; long sleep independently associated with diabetic retinopathy (DR) (OR > 1, P < 0.05); early final awakening and short sleep independently associated with cardiovascular disease (OR > 1, P < 0.05); short sleep independently associated with peripheral arterial disease (OR > 1, P < 0.05); there was no association between poor sleep complaints and neuropathy (P > 0.05).

**Conclusions:**

The study suggests that the poor sleep complaints were distinguishably associated with diabetic vascular complications. Clinicians should take poor sleep complaints into account in diabetes treatment.

## Background

In recent years, evidences from experimental and epidemiologic studies have demonstrated that poor sleep quality is associated with a great deal of health outcomes such as diabetes, cardiovascular disease, hypertension and obesity [1–4]. Poor sleep or sleep disturbances are well-known symptoms in type 2 diabetes, which have been noted by many researchers [5–7]. Diabetic vascular complications covered macrovascular complications including cardiovascular disease (CVD), peripheral arterial disease (PAD) and stroke, and microvascular complications such as diabetic kidney diseases (DKD), neuropathy and retinopathy [8]. Some literatures reported that sleep disturbances or obstructive sleep apnea is one of the risk factors causing CVD [9–11]. One study demonstrated that abnormal sleep patterns could influence the diabetic microvascular complications, in particular diabetic neuropathy [12]. Another study described a novel independent association between diabetic peripheral neuropathy and obstructive sleep apnea (OSA) [13]. Some studies proposed that OSA may contribute to development of DKD [14, 15]. Nishimura et al. concluded that there was an independent relationship between severity of sleep disordered breathing and retinopathy in patients with diabetes [16]. In addition, diabetic vascular complications were considered to have important impact on sleep quality, especially retinopathy and neuropathy [17].

So far, most studies related to sleep disorders and diabetic vascular complications have focused mainly on OSA. There are few studies on various poor sleep complaints with the complications systematically. The aim of the present study was to investigate the association between poor sleep complaints with the diabetic vascular complications in a population-based cross-sectional study.

## Methods

### Materials and subjects

We conducted a population-based cross-sectional study in Chinese adults with type 2 diabetes. Patients were recruited between January 2013 and January 2016 from Metabolic Diseases Hospital of Tianjin Medical University. Inclusion criterion was those patients who were diagnosed with type 2 diabetes according to the 1999 WHO diagnostic criteria for diabetes. Exclusion criteria included type 1 diabetes mellitus, pre-diabetes, diabetes with snoring, acute complications, severe kidney dysfunction, decompensated liver cirrhosis, heart failure with an ejection fraction <30% or NYHA classification >II. The diseases affecting sleep quality were also excluded, including restless leg syndrome, pruritic skin disorders, mental illness and thyroid disease. Consent was obtained and this study design was approved by the local ethics committee and was in accordance with the Declaration of Helsinki. Demographic data such as age and gender were collected and physical examinations including body mass index (BMI) and blood pressure were performed in the subjects. Glycosylated hemoglobin (HbA1C) was measured using standard high-performance liquid chromatography.

### Definitions

The poor sleep complaints included difficulty falling asleep, early final awakening, short sleep and long sleep in this study. The frequency of difficulty falling asleep more than three times a week was defined difficulty falling asleep. The definition of early final awakening was consistent with the above. Sleep duration <6 h/night was defined as short sleep, and sleep duration >9 h/night was defined as long sleep.

Diabetic vascular complications covered microangiopathy such as DKD, DR and diabetic neuropathy, and macroangiopathy such as CVD and lower extremity PAD. DKD was defined as the presence of albuminuria [urinary levels of 24-h microalbumin (UMA) >30 mg/24 h] and/or an estimated glomerular filtration rate (eGFR) <60 mL/min/1.73 m^2^ [15]. eGFR was calculated using the four-variable Modification of Diet in Renal Disease equation (MDRD). Diabetic neuropathy diagnosis based on the results of nerve conduction study, which is a reliable, accurate and sensitive method to evaluate peripheral nerve function [18]. Patients’ clinical symptoms included hyperesthesia, paresthesia, motor weakness or polyradiculopathy [17]. DR was determined by experienced ophthalmologists using dilated indirect ophthalmoscopy or retinal photographs. Retinopathy was defined as at least two microaneurysms and/or retinal hemorrhage and/or other signs of retinal damage [19]. In this study, CVD includes coronary artery disease, hypertensive heart disease, and congestive heart failure (an ejection fraction ≥30% or NYHA classification ≤II) [9]. PAD was diagnosed according to the four typical clinical staging of symptoms, including lower extremity arterial pulses weakening or disappearing found in physical examination and change in lower extremity nutrition disorder or pale, combined with related risk factors such as diabetes, dyslipidemia, hypertension, age and so on. Patients with PAD were confirmed to have different degree of lower extremity artery occlusion using Vascular Ultrasonography. Dyslipidemia was defined that TG, LDL or VLDL was higher than the upper limit of normal or HDL was lower than the lower limit of normal.

### Statistical analysis

All parametrically distributed date are expressed as mean ± standard deviation (SD). Categorical variables are shown as frequencies; Chi square test was used to analyze these variables. Two-sided values of P < 0.05 were considered as statistically significant. Multivariate logistic regression analysis was performed to assess the association between poor sleep complaints with vascular complications using odds ratio (OR) and 95% confidence intervals (CI). We adjusted for major confounding factors expected to affect these relationships (i.e. gender, age, smoking history, BMI, SBP, duration of diabetes, HbA1C and dyslipidemia). SPSS (Statistical Package for Social Sciences) for Windows 18.0 program was used for statistical analysis.

## Results

Table 1 shows the association of poor sleep complaints and baseline characteristics with complications in participants with diabetes. The frequency of early final awakening and short sleep were higher in patients with DKD (56.7 vs. 47.7%, P = 0.005; 51.7 vs. 45.3%, P = 0.043). The frequency of early final awakening and long sleep were higher in patients with DR (55.6 vs. 47.7%, P = 0.011; 6.0 vs.2.2%, P = 0.002). Subjects with diabetic neuropathy had higher prevalence of difficulty falling asleep (30.3 vs. 24.0%, P = 0.020), early final awakening (54.3 vs. 43.7%, P = 0.000) and short sleep (49.5 vs. 43.2%, P = 0.037), respectively. The frequency of early final awakening and short sleep were higher in the diabetic patients with CVD (56.4 vs. 41.5%, P = 0.000; 53.0 vs. 38.6%, P = 0.000, respectively). The frequency of early final awakening and short sleep were higher in the diabetic patients with PAD (52.5 vs. 46.0%, P = 0.043; 49.5 vs. 40.9%, P = 0.007). The patients with DKD had higher BMI, SBP, DBP and HbA1C (P < 0.05), longer diabetes duration (P < 0.05), and higher rate of male (60.9 vs. 51.9%, P < 0.05) compared to those without DKD. The patients with DR were older than those without DR, and had higher rate of female (51.7 vs. 42.0%, P < 0.05), longer diabetes duration (P < 0.05), higher SBP (P < 0.05). The patients with neuropathy were older (P < 0.05), and had longer diabetes duration (P < 0.05), higher SBP (P < 0.05) and lower HbA1C (P < 0.05), compared to those without neuropathy. The patients with CVD were older (P < 0.05), and had higher BMI, SBP (P < 0.05), lower HbA1C (P < 0.05), longer diabetes duration (P < 0.05) compared to those without CVD. The patients with PAD were older (P < 0.05), and higher rate of male (56.2 vs. 48.4%, P < 0.05), longer diabetes duration (P < 0.05), lower HbA1C (P < 0.05) compared to those without PAD.

**Table 1:** Associations of poor sleep complaints and baseline characteristics with complications in subjects with type 2 diabetes mellitus

Microangiopathies	DKD			Retinopathy			Neuropathy		
	Absent	Present	P value	Absent	Present	P value	Absent	Present	P value
Complaints of poor sleep									
Difficulty fall asleep	232 (28.1)	98 (27.2)	0.778	208 (26.8)	124 (30.0)	0.250	110 (24.0)	224 (30.3)	*0.020*
Early awake	394 (47.7)	204 (56.7)	*0.005*	370 (47.7)	230 (55.6)	*0.011*	200 (43.7)	402 (54.3)	*0.000*
Short sleep <6 h	374 (45.3)	186 (51.7)	*0.043*	372 (47.9)	186 (44.9)	0.330	198 (43.2)	366 (49.5)	*0.037*
Long sleep >9 h	22 (2.8)	18 (5.2)	0.057	16 (2.2)	24 (6.0)	*0.002*	16 (3.6)	24 (3.5)	1.000
Male (%)	402 (51.9%)	212 (60.9%)	*0.005*	420 (58.0%)	194 (48.3%)	*0.002*	362 (52.5%)	258 (58.1%)	0.067
Age (years)	56.13 ± 11.20	57.21 ± 11.63	0.149	55.75 ± 11.72	57.78 ± 10.41	*0.004*	52.64 ± 12.63	58.91 ± 9.63	*0.000*
BMI (kg/m^2^)	26.17 ± 3.95	27.34 ± 3.71	*0.000*	26.53 ± 4.01	26.43 ± 3.77	0.690	26.59 ± 4.06	26.47 ± 3.83	0.639
Diabetes duration (years)	8.18 ± 7.26	10.68 ± 7.38	*0.000*	7.19 ± 6.75	12.13 ± 7.54	*0.000*	5.48 ± 6.27	11.20 ± 7.23	*0.000*
SBP (mmHg)	130.63 ± 14.01	136.06 ± 17.57	*0.000*	131.29 ± 14.8	134.65 ± 16.2	*0.005*	130.94 ± 15.4	133.15 ± 15.27	*0.019*
DBP (mmHg)	80.01 ± 8.51	81.57 ± 9.75	*0.007*	80.42 ± 8.70	80.52 ± 9.32	0.870	80.90 ± 9.23	80.22 ± 8.19	0.212
HbA1C (%)	8.96 ± 1.79	9.23 ± 1.70	*0.021*	9.05 ± 1.89	9.06 ± 1.51	0.927	9.29 ± 1.94	8.88 ± 1.62	*0.000*

Macroangiopathies	CVD			PAD					
	Absent	Present	P value	Absent	Present	P value			
Complaints of poor sleep									
Difficulty fall asleep	132 (26.8)	202 (28.8)	0.472	90 (25.6)	256 (29.5)	0.183			
Early awake	204 (41.5)	396 (56.4)	*0.000*	162 (46.0)	456 (52.5)	*0.043*			
Short sleep <6 h	190 (38.6)	372 (53.0)	*0.000*	144 (40.9)	430 (49.5)	*0.007*			
Long sleep >9 h	18 (3.9)	22 (3.3)	0.624	10 (4.1)	30 (3.4)	0.562			
Male (%)	264 (57.6%)	354 (52.7%)	0.101	118 (48.4%)	496 (56.2%)	*0.030*			
Age (years)	52.20 ± 11.96	59.33 ± 9.90	*0.000*	51.71 ± 12.95	57.79 ± 10.43	*0.000*			
BMI (kg/m^2^)	25.99 ± 3.87	26.87 ± 3.91	*0.000*	26.44 ± 4.12	26.51 ± 3.85	0.828			
Diabetes duration (years)	7.27 ± 6.99	10.07 ± 7.49	*0.000*	6.61 ± 6.40	9.61 ± 7.56	*0.000*			
SBP (mmHg)	129.80 ± 14.3	133.93 ± 15.5	*0.000*	131.93 ± 16.4	132.38 ± 15.1	0.699			
SBP (mmHg)	80.65 ± 8.52	80.35 ± 9.18	0.589	80.76 ± 9.52	80.38 ± 8.76	0.572			
HbA1C (%)	9.42 ± 1.99	8.80 ± 1.55	*0.000*	9.48 ± 1.98	8.93 ± 1.69	*0.000*			

Italics values are statistically significant

*DKD* diabetic kidney disease; *CVD* cardiovascular disease; *PAD* peripheral arterial disease; *BMI* body mass index; *SBP* systolic blood pressure; *DBP* diastolic blood pressure; *PSQI* Pittsburgh Sleep Quality Index; *HbAlc* glycosylated hemoglobin type A1c

Table 2 describes the effect of the demographics (gender, age and BMI) on the poor sleep complaints. The complaints were individually saved as dependent variable, and the demographics were saved as independent variables. Older patients were more likely to have these complaints, difficulty fall asleep [OR 1.015, 95% CI (1.002–1.027) P = 0.020], early awake [OR 1.045, 95% CI (1.032–1.057) P = 0.000] and short sleep [OR 1.015, 95% CI (1.004–1.026) P = 0.006]. The female were more vulnerable to have difficulty fall asleep [OR 1.764, 95% CI (1.350–2.307) P = 0.000]. The fatter participants were less trouble in falling asleep [OR 0.955, 95% CI (0.922–0.990) P = 0.012].

**Table 2:** Effect of the demographics on the poor sleep complaints

Demographics	Difficulty fall asleep		Early awake		Short sleep		Long sleep	
	OR (95% of CI)	P value	OR (95% of CI)	P value	OR (95% of CI)	P value	OR (95% of CI)	P value
Gender	1.764 (1.350–2.307)	*0.000*	1.079 (0.844–1.378)	0.545	1.199 (0.943–1.523)	0.138	1.559 (0.807–3.013)	0.186
Age	1.015 (1.002–1.027)	*0.020*	1.045 (1.032–1.057)	*0.000*	1.015 (1.004–1.026)	*0.006*	0.965 (0.940–0.991)	*0.008*
BMI	0.955 (0.922–0.990)	*0.012*	0.997 (0.966–1.028)	0.832	1.022 (0.992–1.054)	0.153	1.029 (0.952–1.113)	0.471

The poor sleep complaints were individually saved as dependent variable, and the demographics were saved as independent variable

Italics values are statistically significant

*BMI* body mass index

Table 3 shows the multivariate analysis for impact of poor sleep complaints on diabetic complications in the populations (model 1). In this model, diabetic vascular complications were individually put into dependent variables, and poor sleep complaints were individually put into independent variables. We adjusted for possible factors (age, gender, duration of diabetes, BMI, SBP, HbA1C, smoking history and dyslipidemia) in the regression analysis. Subjects with short sleep had a higher risk of developing DKD [OR 1.317, 95% CI (1.006–1.723) P = 0.045]; participants with long sleep had a higher risk of developing DR [OR 3.409, 95% CI (1.671–6.995) P = 0.001]; the risk of CVD was higher among the subjects with final early awakening [OR 1.348,95% CI (1.042–1.745) P = 0.023] and/or short sleep [OR 1.589, 95% CI (1.232–2.049) P = 0.000); the risk of PAD was higher among the subjects with short sleep [OR 1.343, 95% CI (1.024–1.762) P = 0.033); after adjustment of potential confounders, poor sleep complaints have no association with the development of neuropathy (P > 0.05).

**Table 3:** Multiple logistic regression analysis for impact of poor sleep complaints on vascular complications in subjects with type 2 diabetes mellitus (model 1)

Microangiopathies	DKD		Retinopathy		Neuropathy	
	OR (95% of CI)	P value	OR (95% of CI)	P value	OR (95% of CI)	P value
Poor sleep complaints						
Difficulty fall asleep	1.015 (0.749–1.376)	0.924	0.970 (0.726–1.296)	0.835	1.134 (0.841–1.529)	0.410
Early wake	1.275 (0.966–1.682)	0.086	1.084 (0.831–1.413)	0.554	1.009 (0.771–1.320)	0.948
Short sleep <6 h	1.317 (1.006–1.723)	*0.045*	0.774 (0.597–1.004)	0.054	1.152 (0.886–1.499)	0.291
Long sleep >9 h	1.829 (0.893–3.746)	0.099	3.409 (1.671–6.995)	*0.001*	1.342 (0.636–2.828)	0.440

Macroangiopathies	CVD		PAD	
	OR (95% of CI)	P value	OR (95% of CI)	P value
Poor sleep complaints				
Difficulty fall asleep	0.901 (0.678–1.197)	0.472	1.091 (0.803–1.483)	0.577
Early wake	1.348 (1.042–1.745)	*0.023*	0.967 (0.733–1.277)	0.815
Short sleep <6 h	1.589 (1.232–2.049)	*0.000*	1.343 (1.024–1.762)	*0.033*
Long sleep >9 h	0.947 (0.438–1.976)	0.947	1.034 (0.466–2.294)	0.934

The complications of diabetes were individually put into dependent variables, poor sleep complaints were individually put into independent variables, logistic regression analysis was adjusted for gender, age, duration of diabetes, smoking history, BMI (body mass index), SBP (systolic blood pressure), HbA1C (glycosylated hemoglobin type A1c) and dyslipidemia

Italics values are statistically significant

*DKD* diabetic kidney disease; *CVD* cardiovascular disease; *PAD* peripheral arterial disease

Table 4 shows the multivariate analysis for impact of diabetic complications on poor sleep complaints in the studied-populations (model 2). In the model, poor sleep complaints were individually put into dependent variables, and diabetic vascular complications were individually put into independent variables. We also adjusted for possible factors (age, gender, duration of diabetes, BMI, SBP, HbA1C, smoking history and dyslipidemia) in the regression analysis. CVD (OR 1.364, 95% CI 1.055–1.765, P = 0.018) was independent variable for early final awakening; DKD (OR 1.322, 95% CI 1.011–1.730, P = 0.042), CVD (OR 1.599, 95% CI 1.239–2.063, P = 0.000) and PAD (OR 1.365, 95% CI 1.041–1.790, P = 0.025) were independent variables for short sleep; DR (OR 3.660, 95% CI 1.762–7.601, P = 0.001) was independent variable for long sleep.

**Table 4:** Multiple logistic regression analysis for impact of vascular complications on poor sleep complaints in subjects with type 2 diabetes mellitus (model 2)

Variables	Difficulty fall asleep		Early wake		Short sleep		Long sleep	
	OR (95% of CI)	P value	OR (95% of CI)	P value	OR (95% of CI)	P value	OR (95% of CI)	P value
DKD	1.052 (0.778–1.422)	0.741	1.300 (0.986–1.713)	0.063	1.322 (1.011–1.730)	*0.042*	1.892 (0.945–3.788)	0.072
Retinopathy	0.983 (0.738–1.310)	0.909	1.073 (0.822–1.399)	0.605	0.780 (0.601–1.011)	0.061	3.660 (1.762–7.601)	*0.001*
Neuropathy	1.149 (0.854–1.545)	0.359	1.022 (0.782–1.338)	0.871	1.160 (0.893–1.509)	0.267	1.511 (0.694–3.289)	0.298
CVD	0.896 (0.675–1.190)	0.450	1.364 (1.055–1.765)	*0.018*	1.599 (1.239–2.063)	*0.000*	1.008 (0.486–2.089)	0.948
PAD	1.110 (0.816–1.509)	0.507	0.982 (0.746–1.294)	0.899	1.365 (1.041–1.790)	*0.025*	1.063 (0.478–2.361)	0.881

Poor sleep complaints were individually put into dependent variables, the complications of diabetes were individually put into independent variables, logistic regression analysis was adjusted for gender, age, duration of diabetes, smoking history, BMI (body mass index), SBP (systolic blood pressure), HbA1C (glycosylated hemoglobin type A1c) and dyslipidemia

Italics values are statistically significant

*DKD* diabetic kidney disease; *CVD* cardiovascular disease; *PAD* peripheral arterial disease

## Discussion

Sleep disturbances, a condition with important for hypertension, diabetes and cardiovascular disease [20–22], has been less researched for diabetes-associated vascular diseases. In addition, current studies related to sleep and diabetic vascular complications have focused mainly on OSA [15, 23–25]. Our work differs from previous studies by offering exploration of the association between various poor sleep complaints and vascular complications in diabetes. Such research is particularly important, which are showing rapidly increasing prevalence of poor sleep complaints and diabetes in populations.

In this study, we described the effect of demographics on sleep complaints of the recruited participants. As expected, older patients were more likely to have these sleep complaints, difficulty fall asleep, early awake and short sleep; the female have a harder time falling asleep; the fatter participants were less trouble in falling asleep. From our data (Table 1), we found that age, gender, BMI, SBP, DBP, diabetes duration and HbA1C have impact on vascular disease distinguishably. Therefore, these factors were all adjusted in logistic regressions.

Interestingly, we demonstrated that early final awakening and short sleep both were risk factors for DKD from Chi square test. However, only short sleep persisted after the adjustment for a wide range of demographic and clinically relevant confounders in logistic regression analysis (model 1). Even though sleep disturbances or OSA has been proved as risk factor for development and progression of DKD [15, 26], short sleep may play great role in pathogenesis of DKD from this research. Meanwhile, we found that diabetic patients with DKD had shorter sleep duration in logistic regression (model 2), demonstrating that DKD and short sleep were interrelated closely.

Our data revealed early final awakening and long sleep were risk factors for DR from Chi square test. But only long sleep still was risk factors for DR after the adjustment in logistic regression (model 1). There was a literature reporting that short and long sleep was associated with high prevalence of DR in men and they speculated sleep deprivation may be involved in the pathogenesis of DR development [27]. In model 2, we found that DR patients had longer sleep; however, one report said the presence of diabetic retinopathy impairs the sleep quality, sleep latency, sleep duration and diurnal function in diabetic patients [17]. The proportion of participants with long sleep was significantly minor in the study, so we did not consider that long sleep was related to DR.

Tahrani et al. provided evidence that OSA is independently associated with diabetic peripheral neuropathy [13]. There are literatures also reporting that pain due to peripheral neuropathy causes nighttime awakening and this leads to disturbed sleep [17, 28, 29]. But there was no association between poor sleep complaints and diabetic neuropathy from the above regression analysis.

From the previous studies, sleep disturbances and/or short sleep were risk factors for CVD [9, 30–32]. One report proposed that frequent awakenings and early morning awakening were not associated with a significantly increased risk of CVD mortality in older populations [33]. Nevertheless, the prevalence of early final awakening was more in patients with CVD than those without CVD; similar was the trend with short sleep in this study. In the regression analysis model 1, early final awakening and short sleep remained independently associated with CVD after adjustment. In addition, association of CVD with early final awakening and short sleep was showed in the model 2, which have not been reported in diabetes patients to the best of our knowledge.

We tried to assess the association between poor sleep complaints with lower extremity PAD in the type 2 diabetes patients. There was great difference between the patients with and without PAD in the prevalence of early final wakening and short sleep from Chi square test. In the model 1, only short sleep was risk factor for PAD after the adjustment. Diabetic patients with PAD had shorter sleep duration in logistic regression model 2 after adjustment. Even though a few current literatures revealed that prevalence of OSA is high in patients with PAD [34, 35], there is little literature reporting the association between poor sleep complaints and PAD.

Mechanisms of poor sleep complaints impacting morbidity of diabetic vascular complications have been speculated. Pathogenesis of vascular diseases in diabetes ascribe to oxidative stress, low-grade inflammation, endothelial dysfunction and so on [36]. First, poor sleep complaints could activate the autonomic nervous system and elevate the secretion of catecholamine, which can stimulate production of inflammatory mediators and could lead to metabolic imbalance [37]. Second, sleep disturbances and sleep fragmentation could activate pro-inflammatory transcription factors such as nuclear factor kappa B (NF-κ B), which is a key player in inflammatory and innate immune responses and a master regulator of inflammatory gene expression, and genes such as TNF-α or IL-8 that are important to the atherosclerotic process. Sleep disturbances may also aggravate oxidative stress which could sharpen endothelial dysfunction and increase insulin resistance [38].

The strength of this study was that it is the first time to assess the relationship between diabetic complications and poor sleep complaints using validated questionnaire roundly. Secondly, the studied-population was large and representative, so the results were worthy to be trusted and extrapolated. However, some limitations of this study should be taken attention. First, the adjusted-confounders were identical in the regression analysis, and this may lead to inaccurate adjustment for each diabetic complication. Second, it is difficult to draw an exact causal relationship between complications of diabetes and poor sleep complaints due to cross-sectional design of the study. In addition, some sleep complaints such as daytime sleepiness and frequent awakening were not assessed in the study, and we should perfect it in future study.

## Conclusions

Poor sleep complaints were distinguishably associated with diabetic vascular complications. Larger prospective trails using the objective assessment by polysomnography are warranted to investigate the underlying mechanisms. Clinicians should pay more attention to poor sleep complaints for improving diabetes patients life quality.

## Abbreviations

T2DM: type 2 diabetes mellitus
PSQI: Pittsburgh sleep quality index
DKD: diabetic kidney disease
DR: diabetic retinopathy
CVD: cardiovascular disease
PAD: peripheral arterial disease
